# Preparation of Chitosan Nanocompositeswith a Macroporous Structure by Unidirectional Freezing and Subsequent Freeze-Drying

**DOI:** 10.3390/md12115619

**Published:** 2014-11-24

**Authors:** Inmaculada Aranaz, María C. Gutiérrez, María Luisa Ferrer, Francisco del Monte

**Affiliations:** Instituto de Ciencia de Materiales de Madrid (ICMM), Consejo Superior de Investigaciones Científicas (CSIC), (Materials Science Institute of Madrid, Spanish National Research Counsil), Cantoblanco 28049, Madrid, Spain; E-Mails: mcgutierrez@icmm.csic.es (M.C.G.); mferrer@icmm.csic.es (M.L.F.)

**Keywords:** chitosan, carbon nanotubes, nanocomposites, scaffolds, cryogels, mineralization, electrodeposition, drug delivery, tissue engineering, fuel cells

## Abstract

Chitosan is the *N*-deacetylated derivative of chitin, a naturally abundant mucopolysaccharide that consists of 2-acetamido-2-deoxy-β-d-glucose through a β (1→4) linkage and is found in nature as the supporting material of crustaceans, insects, *etc.* Chitosan has been strongly recommended as a suitable functional material because of its excellent biocompatibility, biodegradability, non-toxicity, and adsorption properties. Boosting all these excellent properties to obtain unprecedented performances requires the core competences of materials chemists to design and develop novel processing strategies that ultimately allow tailoring the structure and/or the composition of the resulting chitosan-based materials. For instance, the preparation of macroporous materials is challenging in catalysis, biocatalysis and biomedicine, because the resulting materials will offer a desirable combination of high internal reactive surface area and straightforward molecular transport through broad “highways” leading to such a surface. Moreover, chitosan-based composites made of two or more distinct components will produce structural or functional properties not present in materials composed of one single component. Our group has been working lately on cryogenic processes based on the unidirectional freezing of water slurries and/or hydrogels, the subsequent freeze-drying of which produce macroporous materials with a well-patterned structure. We have applied this process to different gels and colloidal suspensions of inorganic, organic, and hybrid materials. In this review, we will describe the application of the process to chitosan solutions and gels typically containing a second component (e.g., metal and ceramic nanoparticles, or carbon nanotubes) for the formation of chitosan nanocomposites with a macroporous structure. We will also discuss the role played by this tailored composition and structure in the ultimate performance of these materials.

## 1. Introduction

The vast majority of processes devised so far for the achievement of foams and scaffolds (phase emulsion, air bubbling, or use of templates, among others) make use of solvents and templates [1,2,3,4,5]. The complete removal of these chemical compounds is typically required prior to the use of the resulting materials in many applications—e.g., biocatalytical/catalytical or biomedical ones, among others. For instance, the absence of undesired byproducts can be of help in either reducing nanoparticles poisoning in catalytic/biocatalytic reactions or preventing denaturation of biological entities in biomedical applications. Moreover, scaffolds biocompatibility is also improved. Unfortunately, this removal is by no means trivial in most of cases so the search for chemical routes that are template-free or, at least, make use of friendly templates is challenging. Within this context, cryogenic processes offer an interesting alternative. The process consists of the freezing of colloidal aqueous suspensions. The ice formation causes most solutes originally dispersed in the aqueous suspension to be segregated from the ice phase, giving rise to a macroporous structure characterized by “fences” of matter enclosing ice. The scaffolds obtained after subsequent drying (by both simple thawing and freeze-drying) show a macroporosity that corresponds to the empty areas where ice crystals originally resided. Cryogels of polymeric nature were first reported more than 40 years ago, and their properties, rather unusual for polymer gels, soon attracted attention [6,7]. Since then, polymer cryogels of many different compositions (e.g., poly(l-lactic acid) and poly(d,l-lactic-co-glycolic acid), gelatin, g-PGA/chitosan, collagen and elastin, collagen-glycosaminoglycan, or albumin-cross-linked polyvinylpyrrolidone (PVP) hydrogels, among others have been widely used in biomedicine (e.g., for tissue engineering and drug delivery purposes) most likely because of the biocompatible character of the process [8,9,10,11,12,13,14,15]. It is worth noting that the process starts from an aqueous solution/suspension or from a hydrogel and proceeds in the absence of further chemical reactions or purification procedures. Actually, the template is just frozen water, the removal of which can be easily accomplished by freeze-drying, thus avoiding potential complications associated with the presence of byproducts or the use of harsh chemical processes for template removal.

**Figure 1:**
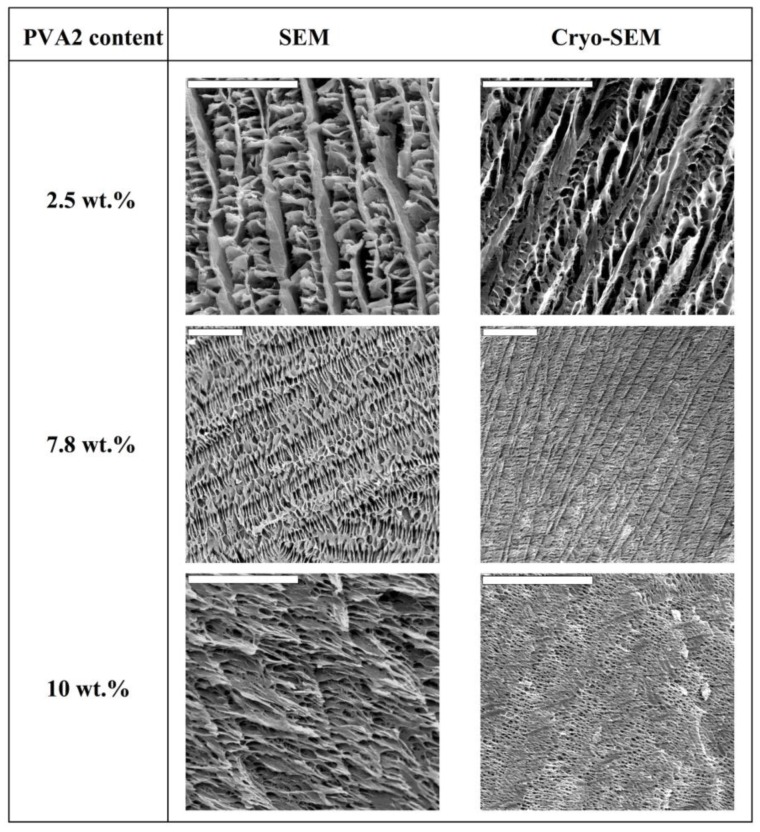
SEM (left) and cryo-etch-SEM (right column) images of cross-sectioned (perpendicular to the direction of freezing) monolithic PVA scaffolds. The freezing rate was 5.9 mm/min and the average molecular weight of the PVA was 72,000 (PVA2) for every ISISA-processed sample. All scale bars are 20 µm. Note that, in some cases, the length of the scale bar changes for better visualization of the scaffold macrostructure. Reprinted with permission from [31]. Copyright ©2007 Wiley-VCH.Verlag.

More recently, our group and some other ones have reported the preparation of inorganic, organic, and hybrid materials with well-patterned macroporous structures via the unidirectional freezing of aqueous suspensions and hydrogels in liquid nitrogen—we coined the term ISISA (ice segregation induced self-assembly) to refer to this process [16,17,18,19,20,21,22,23,24,25,26]. This unidirectional freezing allowed for excellent control—by the freezing conditions, such as the freezing temperatures and/or the sample immersion direction (unidirectional) into the cryogenic liquid, among others—of the morphology of the macroporous structure [27,28,29,30]. Moreover, we have recently demonstrated that the morphology of polyvinylalcohol (PVA) scaffolds can be tailored—in terms of pore diameter, surface area and thickness of matter accumulated between adjacent microchannels—by the averaged molecular weight of polymer, its concentration in the solution, and the freezing rate of the polymer solution [31]. For instance, SEM micrographs of PVA scaffolds prepared from solutions with a low PVA content (*ca.* 2.5 wt % in water) revealed a morphology consisting of poorly interconnected PVA sheets arranged in parallel layers (Figure 1). The increase of the PVA content (*ca.* 7.8 wt % in water) favored the formation of pillars crossing between layers, fully interconnecting the 3D structure. Besides, the size of the porous channels was scaled down, as a consequence of the increased difficulty for the ice crystals to form in the presence of impurities (in this particular case, PVA). A further increase of the PVA content resulted in structures where the porous channels were almost closed; that is, the PVA content reached values that favor the formation of amorphous (supercooled water) rather than crystalline ice, so that no segregation of matter occurred.

**Figure 2:**
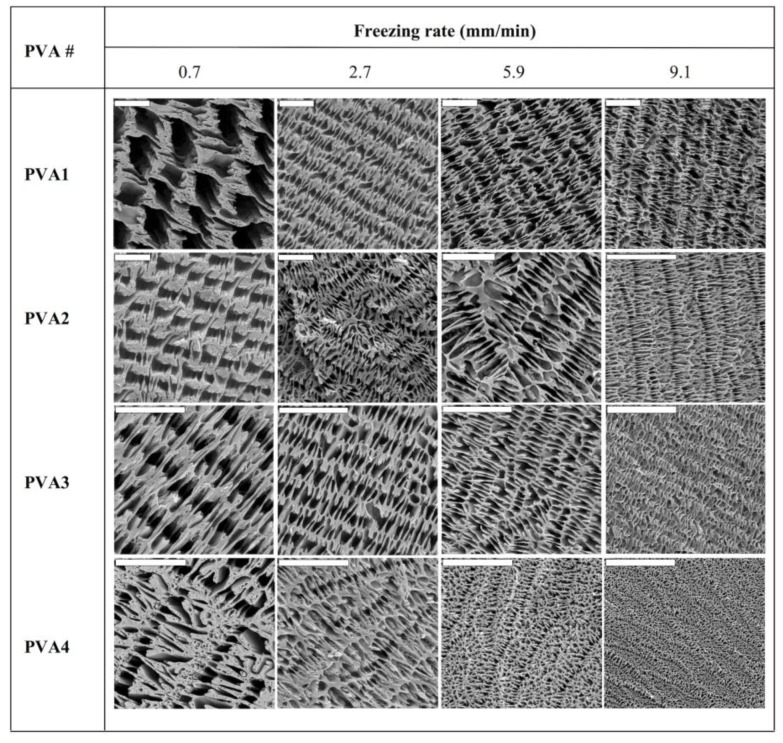
SEM images of cross-sectioned (perpendicular to the direction of freezing) monolithic PVA scaffolds. Tailored morphologies were obtained by using PVA with different weight-average molecular weights (PVA1 = 13,000–23,000, PVA2 = 72,000, PVA3 = 89,000–98,000, and PVA4 = 130,000) and by processing the PVA solution at different freezing rates. All scale bars are 20 µm. Note that, in some cases, the length of the scale bar changes for better visualization of the scaffold macrostructure. The PVA content was 7.8 wt % for every sample. Reprinted with permission from [31]. Copyright ©2007 Wiley-VCH.Verlag.

Nonetheless, low PVA contents (e.g., 2.5 wt % in water) resulted in scaffolds with poor mechanical properties. Thus, we fixed the PVA content at 7.8 wt % for the study of the next variables for tailoring of the morphology: the freezing rate (e.g., 0.7, 2.7, 5.9, and 9.1 mm/min) and the molecular weight of the PVA (PVA1: 13,000–23,000, PVA2: 72,000, PVA3: 89,000–98,000, and PVA4: 130,000, respectively). SEM images revealed that for every sample, the macrostructure resulting from ISISA was characterized by well-aligned micrometer-sized pores in the freezing direction (Figure 2). However, the channel size was strongly dependent on the molecular weights and the freezing rate. Figure 2 actually reflects the large variety of morphologies that can be obtained modulating both parameters. These SEM images revealed a clear tendency regarding the influence of the controlled variables on the porous channel size; that is, it decreased with an increase of either the freezing rate or the molecular weight. It is stated that slow freezing rates allow the formation of large ice crystals, which ultimately template the microchanneled structure. Meanwhile, fast freezing rates favor supercooling and, hence, impede the formation of large ice crystals, so that the microchanneled structure is scaled down.

**Figure 3:**
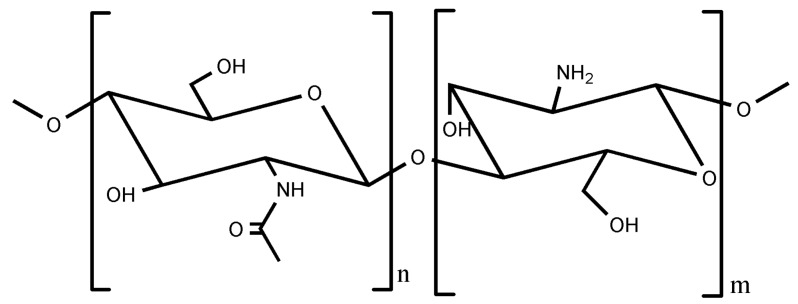
Chemical structure of chitosan where *n* < 40 and *m* > 60.

Within the context of this revision, we would like to mention that the ISISA process can also be applied to polymer of marine origin like chitosan. Chitosan (CHI) is actually a quite interesting polymer because its unique properties—in terms of biocompatibility, non-toxicity and biodegradability —made it very interesting for biomedical and biotechnological applications [32]. The chemical structure of chitosan is shown in Figure 3.

**Figure 4:**
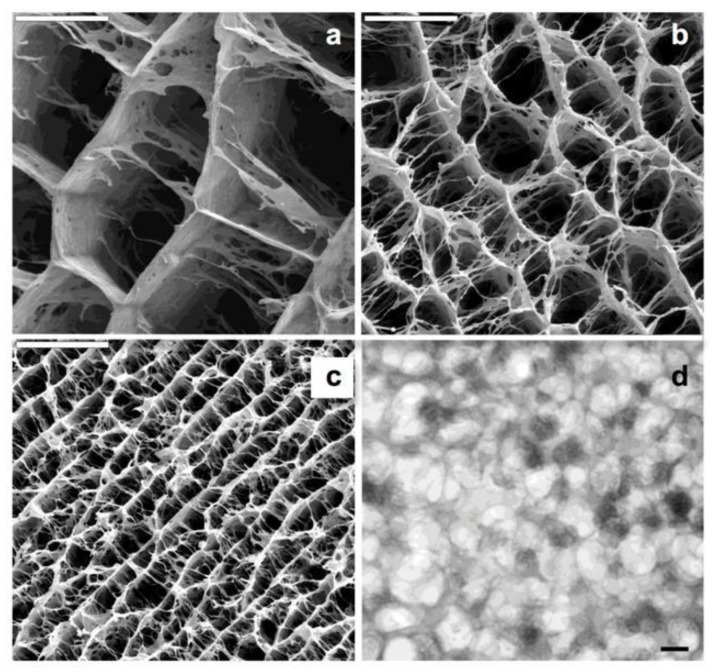
SEM micrographs of different hybrid hierarchical structures resulting from freezing hydrogel nanocomposites, with identical CHI and calcium phosphate composition (93.25 and 6.75 wt %, respectively) at different rates: (**a**) 0.7 mm/min; (**b**) 2.7 mm/min, and (**c**) 5.7 mm/min. Scale bars are 50 μm. TEM (**d**) micrographs of ACP nanoclusters forming the ACP/CHI hierarchical structure. Scale bar is 100 nm. Reprinted with permission from [33]. Copyright ©2008 American Chemical Society.

Chitosan is a polycation whose charge density depends on the degree of acetylation and pH. This macromolecule can dissolve in diluted aqueous acidic solvents due to the protonation of -NH_2_ groups at the C2 position. In acidic conditions, even fully protonated chitosan tends to form aggregates as a result of hydrogen bonds and hydrophobic interactions. This hydrophobic behavior is based on the presence of both the main polysaccharide backbone and the *N*-acetyl groups at C2 position. One can play with the specific pH range where CHI is water-soluble so that its physical gelation can be induced in a controlled fashion. For instance, we may use the urease-assisted hydrolysis of urea to, upon the resulting pH increase, produce CHI gels where the application of the ISISA process creates a homogeneous 3D network structure. Thus, we have applied this process to CHI solutions containing calcium phosphate salts so that we promoted the simultaneous precipitation of calcium phosphate—in form of amorphous calcium phosphate (ACP)—and chitosan gelation to obtain CHI hydrogel nanocomposites [33]. After the application of the ISISA process, the porosity of the hierarchical structure was approximately 85%. The calcination of these materials resulted in the formation of a hierarchical macroporous structure composed of hydroxyapatite nanocrystals (HA-NP). The ISISA process also allowed control of the macroporous size, from 25 up to 90 µm for ACP/chitosan hierarchical structures and from 3 up to 9 μm for HA-NP hierarchical structures. (Figure 4). The mild conditions at which CHI gelation takes place—carried out at biological temperatures of ~37 °C—was critical to obtain microsponge-like morphologies in a homogeneous fashion throughout the whole 3D structure of the resulting scaffolds with superior performances than those of gels obtained by neutralization with alkaline solutions, gaseous NH_3_, or dialyzing chitosan against alkaline media [34]. This combination of features—mild synthesis conditions and tailored structures—opens interesting perspectives for the use of these materials as substrates in tissue engineering applications.

**Figure 5:**
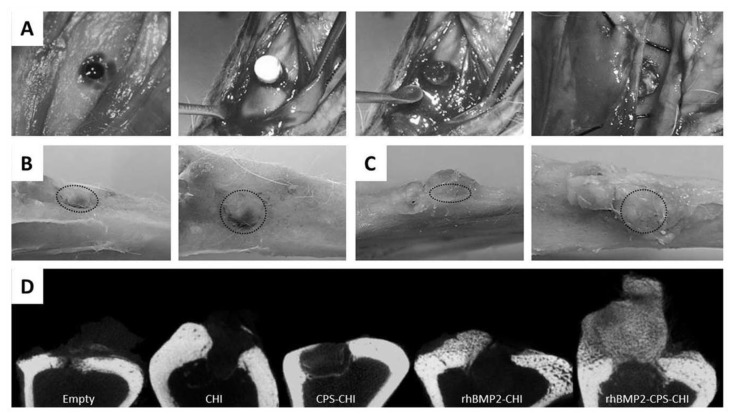
Surgery (**A**), gross morphology after euthanasia (**B**,**C**) and microCT analysis of samples (**D**). Surgery images (A) show scaffold implantation process. After euthanasia gross morphology images were obtained at different angles (B,C) (Dotted circle indicates defect location).Defect area was still observed in CPS-CHI implanted tibias (B), while in rhBMP2-CPS-CHI implanted tibias high amount of newly-formed hard tissue, apparently bone, appeared vertically from the defect (C). MicroCT study (D) confirmed trabecular bone formation in rhBMP2-CPS-CHI implanted tibias, while it seemed no scaffold-resorption in any case. Neither seemed a robust new bone formation in the rest of implanted scaffolds compared to empty controls. Reprinted with permission from [35]. 2014 *PLoS One*. Creative Commons License.

Recently, we have immobilized calcium phosphate salts (CPS) and bone morphogenetic protein 2 (BMP-2)—combined or alone—into chitosan scaffolds using ISISA process [35]. We analyzed whether the immobilized bone morphogenetic protein preserved its osteoinductive capability. We observed that rhBMP2 was not only released in a controlled fashion from CHI scaffolds but also preserved its osteoinductive character after release. Interestingly, we found that this multi-component scaffold exhibited a superior efficacy in bone regeneration than the scaffolds containing only one of the components, either CPS or rhBMP2, separately. This enhanced performance in both osteoconductive and osteoinductive terms opens the path to the future clinical application of these materials in dental surgery and, more specifically, in maxillary sinus augmentation procedure. In this procedure, large area of the maxillary sinus are lifted and replaced with bone, which serves to support future implant placement. It is worth noting that the filling material most used nowadays is porous resorbable hydroxyapatite, which is osteoconductive but not osteoinductive as the rhBMP2-CPS-CHI scaffolds described in this work (Figure 5).

**Figure 6:**
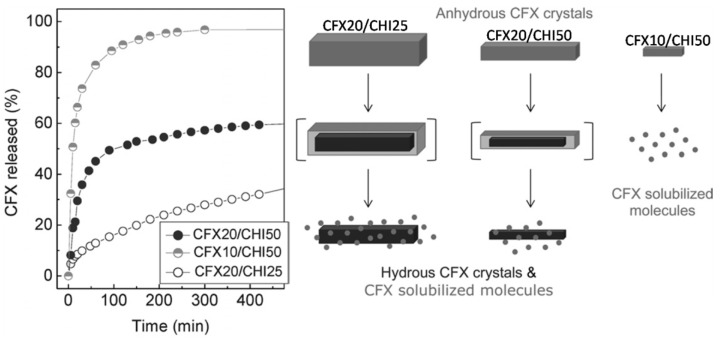
Kinetics release of CFX/CHI scaffolds having different CFX and CHI contents (left). Representation of the solubility (to CFX molecules, light grey)/hydration (to hydrous crystals, dark grey) ratio of anhydrous crystals (light grey) depending on their crystal size (right). Reproduced from [36] with permission from The Royal Society of Chemistry.

Following the same methodology—this is, the gently pH modification of chitosan solutions by the enzymatic hydrolysis of urea-chitosan scaffolds containing ciprofloxacin (CFX, a synthetic fluoroquinolone antimicrobial agent) were produced for drug delivery purposes [36]. In this case, pH modification resulted in both chitosan gelation and CFX crystallization. Thus, a macroporous CHI scaffold containing anhydrous CFX crystals was obtained after submitting the hydrogel to the ISISA process. Interestingly, the kinetic release of CFX in these chitosan scaffolds was controlled by the peculiarities of the anhydrous form in which CFX crystallizes during the ISISA process as well as by the crystals size rather than by the typical mechanisms based on swelling, hydration and/or erosion of the polymer acting as carrier. It is worth noting that CFX may exist in two different crystalline forms, the hydrated and the anhydrous ones. The hydrated form is hardly soluble in water whereas the anhydrous one is very soluble in water and, unless excipients are used, it transforms readily into the hydrated form upon exposure to water [37]. Thus, when anhydrous CFX crystals were exposed to an aqueous environment, there will be a competition between dissolution and hydration according to Figure 6—*i.e.*, first layers of anhydrous crystals will be readily dissolved, but the internal core of anhydrous CFX crystals will become hydrated before dissolution. This is why two kinetics could be easily observed, one burst type due to the dissolution of the external surface of anhydrous CFX crystals, and a second slower-one due to the poor solubility of hydrous CFX crystals. Interestingly, we were able to tune the percentage of CFX released in a burst type fashion just by tailoring the size of the anhydrous CFX crystals and thus, the external surface to internal core ratio of the crystals.

**Figure 7:**
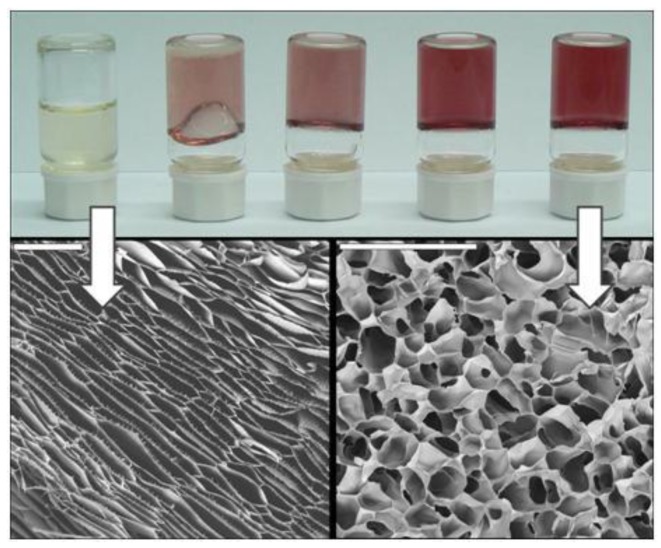
(Top panel) Picture of AuNP-CHI gels having different HAuCl_4_·3H_2_O concentrations (from left to right; nil, 0.2, 0.5, 1, and 2 mM) obtained after thermal treatment at 40 °C over 150 min. Effect of the viscosities and strength hydrogel on scaffold morphology. (Bottom panel) Examples of sols and gels produced by controlling Au concentration lamellar structure from sols (left) and cellular-type morphology (right). Scale bars are 200 µm. Reprinted with permission from [38]. Copyright ©2011 American Chemical Society.

Chitosan gelation could also be induced in presence of gold salts because of the pH increase that results during the transformation of these salts into nanoparticles upon acetic acid consumption [38]. As in the previous cases, the result was the formation of a nanocomposite, in this case composed of a CHI macroporous structure with Au nanoparticles homogeneously distributed throughout the whole 3D structure. In this case, the morphology of the resulting scaffolds was related to either the viscosity or the strength of the solutions or hydrogels subjected to the ISISA process, respectively. Interestingly, both viscosities and strengths increased along with the concentration of the Au salts at the starting solution. Thus, lamellar-type morphologies were obtained in scaffolds prepared from sols, intermediate morphologies were obtained in scaffolds prepared from soft gels, whereas cellular-type morphologies were obtained from strong gels (Figure 7). Interestingly, not only the morphology but also the dissolution and swelling degree of the resulting CHI scaffolds were strongly influenced by the strength of the hydrogels obtained by the *in situ* formation of AuNP.

The capability to, under mild experimental conditions, obtain AuNP-CHI scaffolds with tailored physico-chemical properties without using further chemical additives—this is, the scaffolds were only composed of CHI and AuNPs with neither further reduction nor cross-linking agents–made these materials quite attractive for applications in both catalysis and biomedicine, where the performance of materials can be strongly influenced by the presence of impurities. In particular, we demonstrated the suitability of AuNP-CHI scaffolds for catalytic purposes (e.g., AuNPs assisted reduction of p-nitrophenol by NaBH_4_) with full preservation of the reaction kinetics for up to four cycles as a result of the structural stability gained in AuNPCHI scaffolds.

## 2. Carbon Nanotubes Chitosan Based Scaffolds Produced by Ice Segregation Induced Self-Assembly

Within the context of novel CHI nanocomposites processed in form of macroporous structures, we have also studied those based on carbon nanotubes (CNTs).Since their discovery in 1991, CNTs have been the subject of numerous research works given their unique properties, for example, extremely high electrical conductivities, very high thermal conductivities, and outstanding mechanical properties [39]. As a general trend for any catalytic application, the challenge is the preparation of materials with bimodal porous structures because the combination of high internal reactive surface along the nanostructure with facile molecular transport through broad “highways” would contribute to the performance enhancement [40].

**Figure 8:**
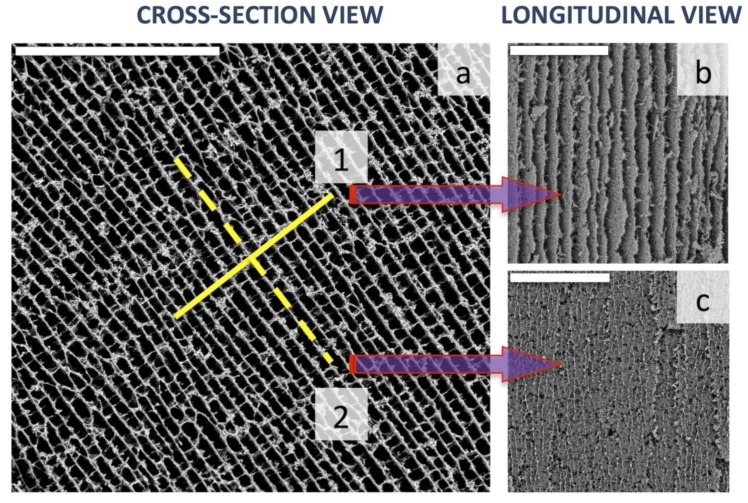
SEM micrographs of MWCNT/CHI scaffolds: (**a**) cross-section view; (**b**) longitudinal view according to plane 1 and (**c**) longitudinal view according to plane 2. Scale bars are 200 µm in every case. Reprinted with permission from [41]. Copyright ©2012 Wiley-VCH.Verlag.

Chitosan is an efficient dispersion agent for carbon nanotubes. Thus, we dispersed multi wall carbon nanotubes (MWCNT) homogenously into a CHI aqueous solution and submitted it to the ISISA process [25]. The resulting materials were highly porous monoliths (specific gravity ~10^−2^) with different shapes (both regular and irregular) and sizes, the 3D structure of which was assembled thanks to the gluing features of CHI. The macroporous architecture was chamber-like, in the form of interconnected MWCNT/CHI sheets arranged in parallel layers (Figure 8). The morphology of the resulting structure was strongly dependent on the MWCNTs wt % at the starting aqueous suspension. Thus, the interconnection of MWCNT/CHI sheets was favored for high MWCNTs contents because of the formation of pillars crossing between layers whereas these pillars were scarcer for low MWCNTs contents. Morphology control was also exerted through the freezing rate but further improvement in the regularity of the patterned macrostructure was not achieved in this particular case. In any case, the ISISA process provided well-aligned microchannels in the direction of freezing (Figure 8) and it is worth noting that MWCNTs were not aligned but indeed percolated throughout the 3D structure.

All these morphological features provided interesting properties to these materials. For instance, the electrical conductivity—as measured by the four-probe method—reached values similar to those obtained for pills of densely packed MWCNTs—e.g., 2.5 S/cm was reached with a monolith containing 89% MWCNTs after freeze-drying. This result encouraged us to use these macroporous MWCNT/CHI nanocomposites as 3D electrodes. Thus, we evaluated their performance as anodes in a direct methanol fuel cell. For this purpose, we used MWCNTs that were surface decorated with Pt nanoparticles prior to their suspension in the CHI aqueous solution. The resulting Pt/MWCNT/CHI 3D architectures allowed for a remarkable improvement (e.g., current densities of up to 242 mA/cm) of the catalytic activity toward the methanol oxidation thanks to efficient fuel and product diffusion through the aligned microchannels of the 3D structure.

**Figure 9:**
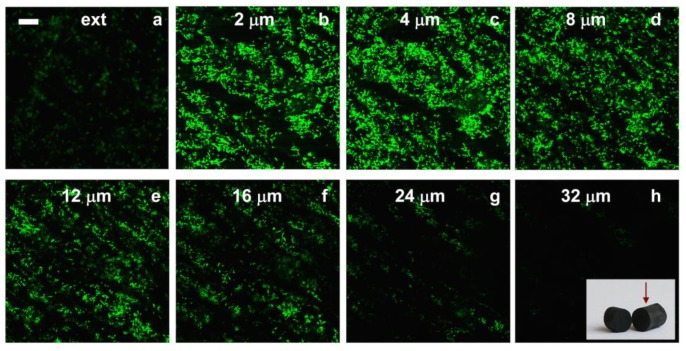
Confocal fluorescence microscope images showing a MWCNT scaffold soaked for 24 h in a suspension of bacteria in culture medium. The depth of focus was (**a**) the external surface and (**b**) 2; (**c**) 4; (**d**) 8; (**e**) 12; (**f**) 16; (**g**) 24 and (**h**) 32 µm. Scale bar is 20 µm. Inset: MWCNT scaffold, where the arrow indicates the view angle used for confocal fluorescent micrographs. Reproduced from [26] with permission from The Royal Society of Chemistry.

We further evaluated the suitability of these materials as 3D electrodes in different fuel cells, in particular as anodes in a microbial fuel cell (MFC).The main issue for energy production in MFCs is, first and obvious, the use of bacteria—or of any other microorganisms—capable of electron shuttle electrons to a current collector and, second, the formation of biofilms that will allow this electron transfer in an effective fashion. In this case, we used MWCNT/CHI 3D architectures—Pt nanoparticles were not required—and we promoted the biofilm formation on the internal surface of the microchannels [26]. Bacteria colonization of the internal structure was attempted by different means. We first tried the direct soaking of the MWCNT/CHI 3D architectures into a bacteria culture medium. Unfortunately, cell proliferation throughout the whole scaffold structure was impeded using this procedure [42]. Confocal microscopy taken from the external surface up to a depth of 32 mm of the monolithic structure revealed how bacteria proliferation was indeed limited to just a few bacteria layers in depth (up to *ca.* 24 µm). Beyond those layers (deeper than 24–32 µm, images g and h of Figure 9), bacterial population experienced a significant decrease—up to depletion—because of nutrients and oxygen consumption by the outer bacteria.

**Figure 10:**
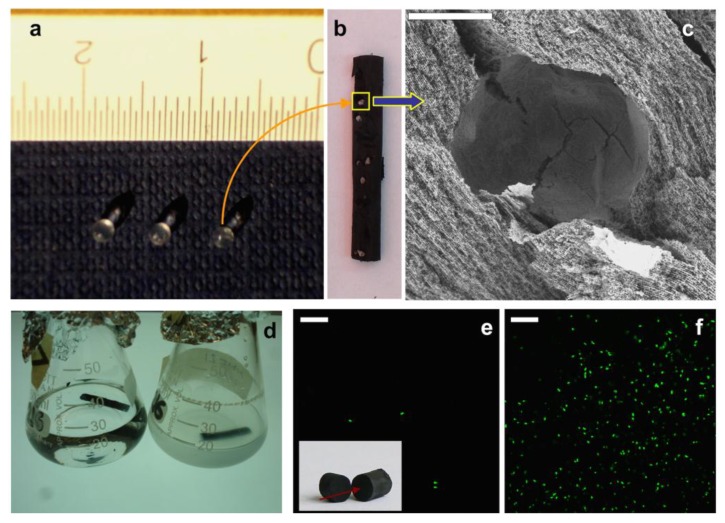
(**a**) Three representative bacteria-glucose-alginate beads with diameters around 1 mm; (**b**) A longitudinal section of a monolithic MWCNT scaffold with immobilized bacteria-glucose-alginate beads, where the homogenous allocation of the beads can be visualized; (**c**) SEM image of a longitudinal section of the MWCNT scaffold with a hole left by a bead (bar is 500 µm); (**d**) MWCNT monoliths with immobilized bacteria-glucose-alginate beads right after soaking in culture medium (left) and after incubation at 37 °C for 24 hours (right); (**e**,**f**) Confocal fluorescence microscope images of the MWCNT monolith with immobilized bacteria-glucose-alginate beads soaked in culture medium before (e) and after (f) incubation at 37 °C for 24 h (bars are 20 µm). Inset: MWCNT scaffold, arrow indicates the view angle used for confocal fluorescent microscopy. In both cases, the focus was at the MWCNT structure and not at the alginate bead. Bacteria visible in (e) most likely corresponded to some minor fraction of bacteria released from alginate. Reproduced from [24] with permission from The Royal Society of Chemistry.

To overcome this partial bacterial colonization of the MWCNT scaffold, we decided to entrap bacteria within beads composed of a natural calcium-alginate polymer (Figure 10a) and add these beads to the MWCNT suspension prior to freezing [24,43]. Unidirectional freezing and subsequent freeze-drying resulted in a monolithic MWCNT scaffold with beads homogeneously immobilized within the three-dimensional macrostructure (Figure 10b,c). The MWCNT scaffolds containing bacteria-glucose-alginate beads were soaked in a culture medium, also containing sodium citrate. Calcium chelation with citrate resulted in dissolution of alginate beads, turning the cavity content to liquid, which allowed for bacterial dispersion and eventual growth within the scaffold. Incubation at 37 °C indeed resulted in bacteria growth and proliferation from the inner to the outer side of the macrostructure up to the culture medium, which became turbid (Figure 10d). Confocal fluorescence microscopy of the scaffold confirmed bacteria proliferation (Figure 10e,f). Unfortunately, the bacterial population per mm^2^—as obtained from the confocal microscope images—was lower than that found in Figure 9 for the MWCNT scaffold monolith soaked in bacteria culture medium (*ca.* 3000 *versus* 39,000 bacteria mm^−2^) and not sufficient for our purposes. There may be several causes of the viability decrease. As in the previous case, limited diffusion of nutrients and oxygen to the inner sides of the scaffold could still play a role. However, the main issue in this case was most likely the cryogenic process followed for scaffold preparation. It is worth noting that freeze-drying processes will unavoidably damage some of the bacterial cells [44].

**Figure 11:**
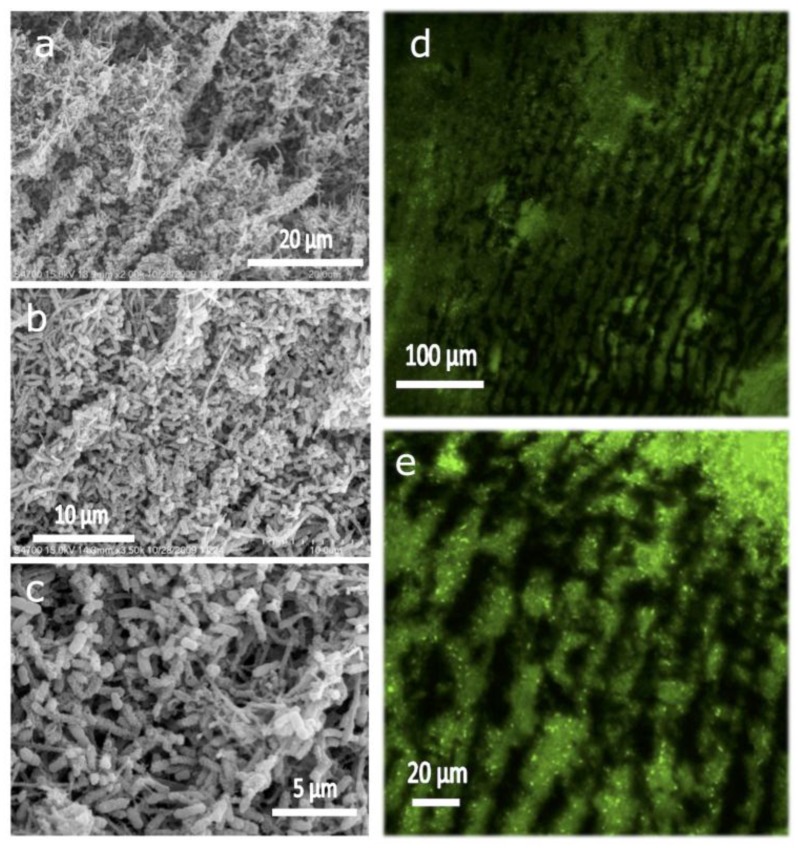
SEM images (**a**–**c**) of biofilm at different magnifications taken from the inner part of the 3D scaffold. CLSM images at different magnifications (**d** and **e**), after staining with live/dead (bacLight) kit, of the longitudinal section of Gs biofilm induced to grow in a 3D scaffold, pre-filled with *Gs*, at 0 V *versus* Ag/AgCl, operated under flow of culture medium (10 mM acetate) for ~320 h. Reprinted with permission from [45]. The Royal Society of Chemistry.

Thus, we explored whether we could force both bacteria and nutrients diffusion through the 3D scaffold [45]. It is worth mentioning that in this case MWCNT scaffolds were submitted to a mild thermal treatment (95 °C over 6 h, similar to that described above for CHI nanocomposites) in order to preserve the integrity of the 3D microchannelled structure during the flow-through experiments. Mass transport was indeed possible in a flow-through configuration thanks to the microchannelled structure of the 3D scaffolds. In this case, we worked with *Geobactersulfurreducens* (*Gs*), a member of the metal-reducing *Geobacteraceae* family, which is capable of complete oxidation of acetate to carbon dioxide with the anode serving as the sole electron acceptor. The scaffold electrode allowed for bacterial colonization of the internal surface area of the 3D structure as well as for mass transport of nutrients and byproducts (Figure 11). Interestingly, the biofilm was capable of providing 19 kA/m, representing the highest volumetric current densities in microbial electrochemical systems reported to date, and a volumetric power density of 2.0 kW/m^3^ in a non-optimized flow MFC configuration.

CNTs have also attracted significant attention in the emerging field of nanobiotechnology, thus being already exploited for the preparation of biosensors, molecular transporters for drug, gene and protein delivery, and fuel-powered artificial muscles [46,47,48]. In the area of tissue engineering, they have been extensively explored for bone regeneration and nerve tissue repair [49,50,51,52,53,54]. In this context, a wide repertoire of methodologies have been explored and optimized for the preparation of 2D CNT-based composites (e.g., films and fibers) [55]. However, the fabrication of 3D architectures has been more rarely accomplished—with some approaches including chitosan-based matrices, gelatin and/or methacrylate hydrogels collagen scaffolds, poly(lactic-co-glycolic acid) or poly(methylmethacrylates)—despite the obvious potentiality of 3D structures in regenerative medicine and tissue engineering because of their capability to mimic the natural extracellular matrix (ECM) in terms of structure, chemical composition, and mechanical properties [41,56,57,58,59,60,61,62,63,64].

However, the toxicity of CNTs remains controversial [65]. In order to bring some insights into the still uncertain interaction of 3D structures composed of CNTs with mammalian cells, we explored the interaction of three cell types (*i.e.*, L929 fibroblasts, Saos-2 osteoblasts and porcine endothelial progenitor cells) with MWCNT/CHI 3D scaffolds that showed different architectural and morphological features at the microscale [66]. For this purpose, the scaffold morphology was modified by (1) the incorporation of MWCNTs with two different lengths and (2) the application of the ISISA process at −65 and at −196 °C. In both cases, we found significant changes in the morphology of the resulting scaffolds in terms of porosity and surface roughness (Table 1).

**Table 1:** Scaffold characterization including porosity, surface roughness, mechanical properties, and electrical conductivity. Adapted from [66] with permission from The Royal Society of Chemistry.

Scaffold	Pore Area per µm^2^ (A_P_)	Pore Width (W_P_) (µm)	RMS Roughness (R_q_) (nm)	Young’s Modulus (MPa)	Conductivity (S/cm)
LNCHI ^a^	0.60 ± 0.14	1.4 ± 2.8	220	5.2	1.70
LNCHI-65 ^b^	0.69 ± 0.05	60 ± 40	190	1.2	0.70
SNCHI ^c^	0.69 ± 0.04	21 ± 4	120	4.0	0.32

^a^ LN: MWCNT length 5–9 µm; ^b^ LNCHI-65: Freezed at −65 °C; ^c^ SN: MWCNT length 3 µm.

**Figure 12:**
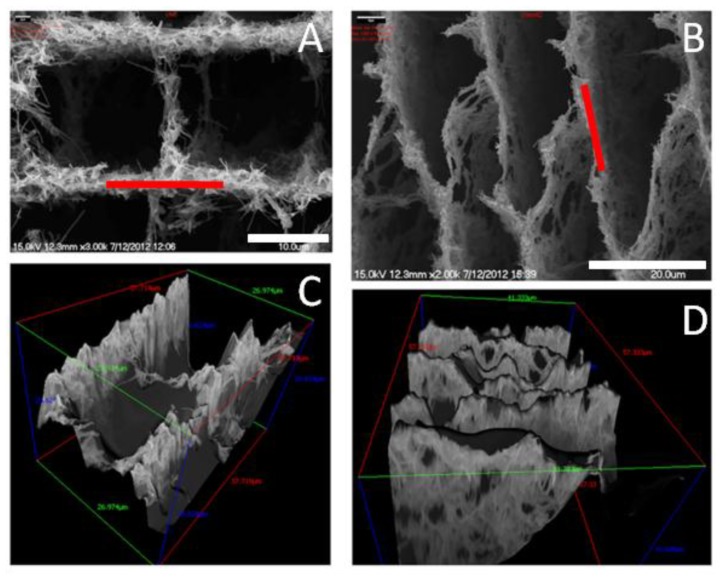
SEM images of LNCHI (**A**) and SNCHI (**B**) scaffolds along with their 3D reconstructed images (**C** and **D**, respectively). Scale bars: 10 mm (A) and 20 mm (B). Red lines in Figure 12 A and B indicate position of measurement. Adapted from [66] with permission from The Royal Society of Chemistry.

We analyzed the pore area per cross-sectioned area (A_P_) and the averaged pore width (W_P_) from SEM micrographs of cross-sectioned scaffolds. The roughness was studied by SEM using a MeX software package for 3D data acquisition and object reconstruction using stereophotogrammetry. Thus, we were capable to obtain the root mean square (RMS) and the roughness (Rq) of the different specimens overa path length of 15 µm. Roughness determination was carried out measuring on the walls that defined the cross-sectioned structures on which cells were cultured (Figure 12).

**Figure 13:**
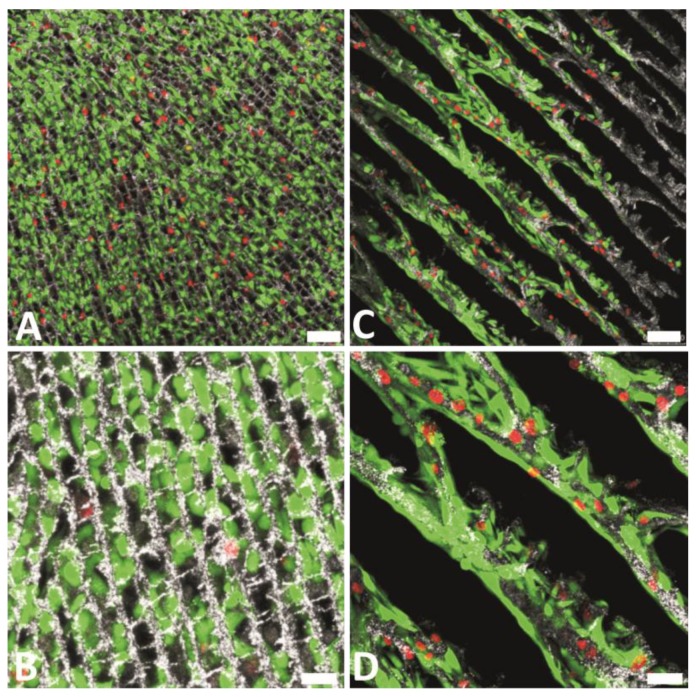
L929 fibroblast cell viability on LNCHI (**A** and **B**), LNCHI-65 (**C** and **D**). Dead cells appear stained in red, while living cells are stained in bright green. Representative culture images at 48 h are shown. Scale bars: 75 mm (**top**) and 25 mm (**bottom**). Reprinted from [66] with permission from The Royal Society of Chemistry.

After determination of the most relevant morphological features of the different scaffolds, we studied cell viability for the three cell types mentioned above using a Live/Dead Viability kit that is based on the fluorescent response of two probes: calcein and ethidiumhomodimer-1 (EthD-1). The cell viability was high in every case although some peculiarities were found for each particular case. For instance, L929 fibroblasts and Saos-2 osteoblasts growth on LNCHI scaffolds occupying pore spaces between scaffold walls so they were basically suspended in the air with just few contact points with the substrate. As compared to L929 and Saos-2 cells, the innate adhesion pattern of porcine endothelial progenitor cells led to the increase of their contact surface with the scaffold. With these latter cells, the substrate roughness played a role in the viability so that SNCHI proved more biocompatible than LNCHI (Figure 13).

**Figure 14:**
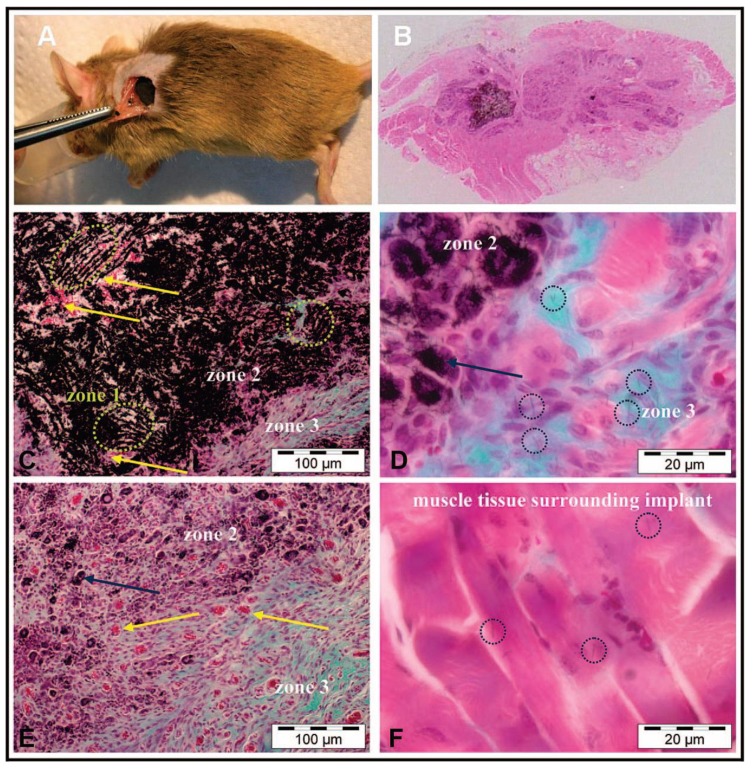
Picture **A** shows a surgical implantation of MWCNT/CHI scaffolds containing adsorbed rhBMP2 into a mouse subcutaneous muscular pocket. Optical micrograph **B** shows regenerated bone tissue and the small fraction of MWCNT/CHI scaffold remaining three weeks after scaffold implantation. Optical micrographs **C**–**F** show details of the three main distinguishable zones observed in micrograph B: Zone 1 where the MWCNT/CHI scaffold structure remained basically intact (marked by green circles), Zone 2 where non-differentiated fibroblasts (purple cells) were colonizing the partially disassembled structure of the MWCNT/CHI scaffold (the positions of representative clusters of MWCNT/CHI aggregates are indicated by blue arrows), and Zone 3 where the MWCNT/CHI scaffold was fully disassembled and individual MWCNT/CHI (the locations of some representative MWCNT/CHI are indicated by blue circles) were dispersed within the regenerated bone tissue (collagen expressing cells are colored blue-green). Note the abundance of blood vessels (containing erythrocytes, of which some representative examples are indicated by yellow arrows) in Zone 2. Optical micrograph **F** shows a few individual MWCNT/CHI (also located within blue circles) dispersed within muscle tissue (colored pink) surrounding the implant. Reprinted with permission from [57]. Copyright ©2007 Elsevier.

The above-described *in vitro* experiments revealed that a case-by-case study is needed to ascertain the biocompatibility of MCWNT/CHI scaffolds. Moreover, one should consider further requirements for *in vivo* experiments where not only biocompatible but also biodegradable materials are required. For this purpose, we studied the ectopic bone formation in muscle tissue by implantation of MWCNT/CHI scaffolds that contained rhBMP-2 (a potent osseo-inductor protein that promotes the differentiation of differentiated cells into an osteoblastic lineage) adsorbed onto the macrostructure [57]. The excellent biocompatibility found in these experiments was ascribed to the lack of metal traces and amorphous carbon in the scaffolds. It is worth noting that, prior to the formation of the scaffold, MWCNT were submitted to a purification process that eliminated any of the typical byproducts coming from MWCNT preparation procedures. Moreover, biocompatibility also benefited from the presence of chitosan that covers partially the surface of the MWCNTs forming the scaffold structure. Pictures shown in Figure 14 allow further discussion with regard to the mechanism governing scaffold degradation and tissue growth. Thus, one could observe at least three different zones. Zone 1 was mostly occupied by MWCNT/CHI scaffold yet resembling its characteristic well-patterned structure. Meanwhile, partial scaffold disassembly at Zone 2 resulted in the presence of agglomerated MWCNT/CHI in a non-ordered fashion. This second zone also exhibited an abundant presence of blood vessels and some few non-differentiated purple cells (e.g., fibroblasts) between the clusters formed by aggregated MWCNT/CHI (Figure 14d). Finally, Zone 3 was completely occupied by collagen-expressing cells (resulting from fibroblasts differentiation in presence of rh-BMP-2 and visualized as blue-green cells) and MWCNT/CHI—neither as a well-patterned structure nor as agglomerates in a nor-ordered fashion—were not longer visualized (Figure 14f). A close inspection to this tissue and to adjacent muscular tissues allowed the visualization of some few non-aggregated MWCNTs. Based on these pictures and subjected to further experiments to corroborate this hypothesis, we concluded that the implanted MWCNT/CHI scaffold underwent a degradation process—concurrent with cell colonization—based first on structure collapse and then in MWCNT dispersion where the individually dispersed MWCNT were most likely incorporated to the blood circulation system and subsequently eliminated through the renal excretion route. 

**Figure 15:**
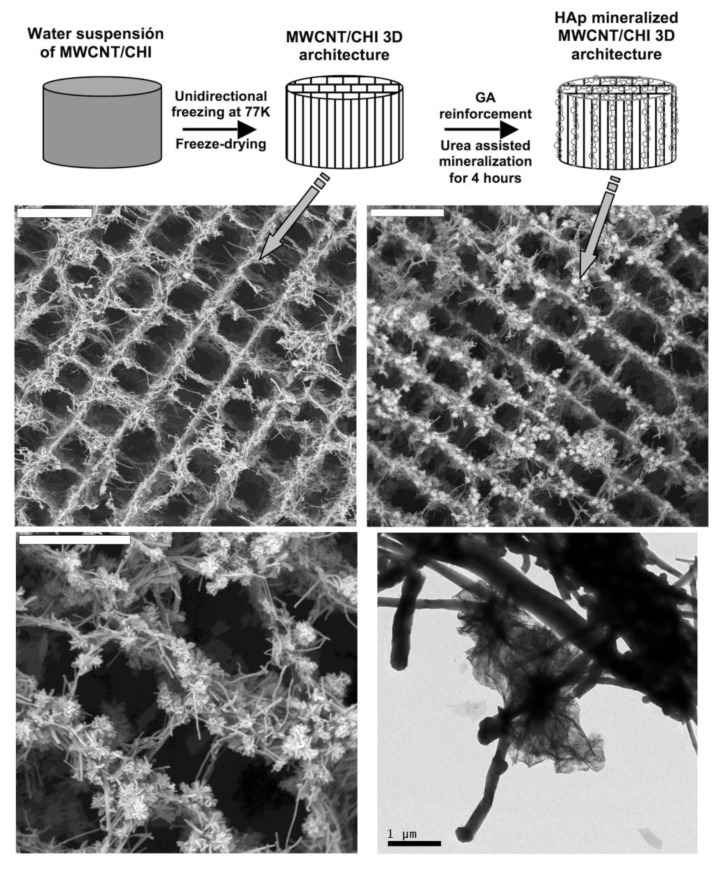
Top panel: Schematic representation of the cryogenic process followed for the preparation of MWCNT scaffolds and of the subsequent processes followed for GA architecture reinforcement and HA mineralization (HA crystals are represented as grease open circles). Middle row: SEM micrographs of MWCNT/CHI scaffolds (left) and Hap mineralized MWCNT/CHI scaffolds (right). Bars are 20 mm. Bottom panel: SEM (left) and TEM (right) micrographs show a detail of Hap crystals on the MWCNT forming the scaffold. Scale bars are 10 and 1 µm, respectively Reproduced from [59] with permission from The Royal Society of Chemistry.

In view of the good osteoinductive response of MWCNT/CHI scaffolds impregnated with rhBMP, we explored the incorporation of other osteoinductive agents like hydroxyapatite (HA). HA mineralization was achieved by impregnation of MWCNT/CHI scaffolds with calcium and phosphate salts, followed by HA precipitation on the scaffold internal structure [59]. HA precipitation was thermally induced upon urea decomposition that was originally incorporated within the scaffold structure besides the calcium and phosphate salts. This process obviously required of certain reinforcement of the scaffold structure, e.g., glutaraldehyde cross-linking. Otherwise, the scaffold would collapse in the acid aqueous solution of calcium and phosphate salts. We found that thermal treatments of 4 hours resulted in the formation of HA crystalline clusters of *ca.* 1 µm in diameter homogeneously distributed throughout the scaffold macrostructure (Figure 15). Further mineralization was attempted upon prolonged thermal treatments but the enlargement of the crystalline clusters (up to 5 µm) disrupted their originally homogeneous distribution throughout the MWCNT/CHI scaffolds structure so that one could find internal surfaces either fully HA-coated or HA depleted.

**Figure 16:**
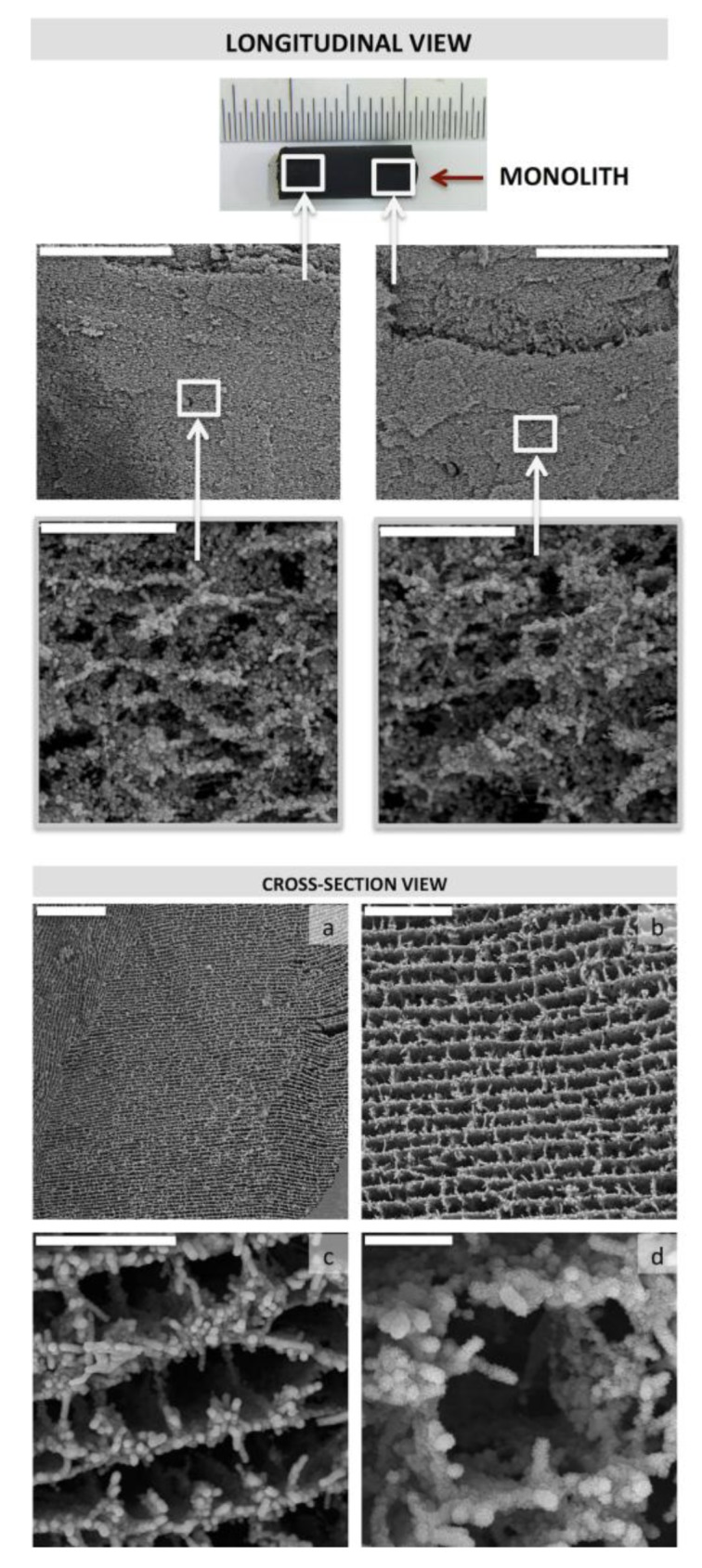
SEM micrographs of MWCNT scaffolds mineralized in flow-through conditions revealed the long-range homogeneity of the mineral coating along the entire scaffold structure of the monolith. Electrodeposition was carried out over 30 min at 30 °C and 1.4 V. Top panel: Longitudinal view, scale bars represent 400 μm (**top**) and 20 μm (bottom).Bottom panel: Cross-Section view, scale bars represent 200 μm (**a**); 50 μm (**b**); 20 μm (**c**); and 5 μm (**d**). Reprinted with permission from [41]. Copyright ©2012 WILEY-VCH Verlag.

This problem was overcome in a subsequent work taking advantage of two particular features, e.g., the electrical conductivity and microchannelled structure—of the MWCNT/CHI scaffolds. Thus, we designed a flow-through system so that the solution containing calcium and phosphate salts was forced to flow throughout the MWCNT/CHI monolithic structure [41] (Figure 16). A layer of crystalline clusters was homogeneously electrodeposited throughout the entire internal surface of the MWCNT/CHI monolithic structure—both longitudinally and radially—by the application of a certain voltage.

**Figure 17:**
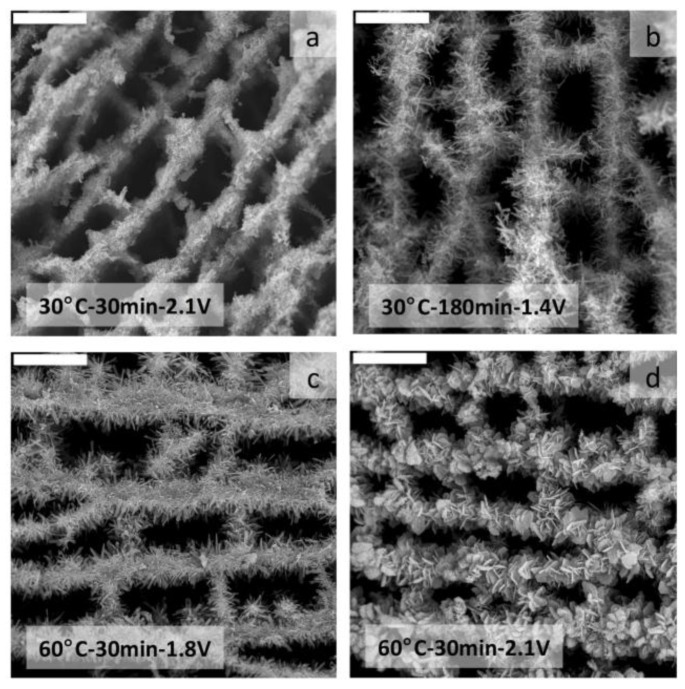
SEM micrographs of MWCNT/CHI scaffolds after mineralization in flow-through conditions that revealed the homogeneity of the mineral coating irrespective of the experimental conditions used for electrodeposition; (**a**) 30 °C-30 min-2.1 V; (**b**) 30 °C-180 min-1.4 V; (**c**) 60 °C-30 min-1.8 V; and (**d**) 60 °C-30 min-2.1 V. Scale bars are 20 µm in every case. Reprinted with permission from [41]. Copyright ©2012 WILEY-VCH. Verlag.

The applied voltage itself, the time the voltage was applied and the temperature at which the voltage was applied determined the nature of the HA crystals, from dicalcium phosphate dehydrate (DCPD) to octacalcium phosphate up to hydroxyapatite (Figure 17). DCPD-mineralized MWCNT/CHI scaffolds showed a remarkable biocompatibility when tested with human osteoblast cells. Interestingly, the initial presence of DCPD on these materials promoted a faster and significant osteoblast terminal differentiation (as early as seven days in calcifying media). Thus, these scaffolds could be considered as attractive multifunctional materials combining a 3D hierarchical structure, biocompatibility and osteoconductive properties.

## 3. Conclusions

In this review, we highlight the ability of ice-templating processes for the preparation of chitosan-based macroporous materials—either with bare chitosan or in form of nanocomposites. The ISISA process allowed us to produce highly porous hybrid materials with high surface area and straightforward molecular transport. Besides and with regard to the composition, the combination between the intrinsic properties of chitosan with those coming from the additional components (organic, inorganic, biomolecules and even microorganisms)offered interesting possibilities in number of applications (e.g., tissue engineering, drug delivery, catalytic applications, energy production, among others) as described along this paper.
